# PTSD and re-offending risk: the mediating role of worry and a negative perception of other people's support

**DOI:** 10.3402/ejpt.v4i0.21382

**Published:** 2013-12-20

**Authors:** Vittoria Ardino, Luca Milani, Paola Di Blasio

**Affiliations:** 1 PSSRU Unit, Department of Social Policy, London School of Economics and Political Science, London, UK; 2 C.R*i.d.e.e*, Department of Psychology, Catholic University of Milan, Italy; 3 C.R*i.d.e.e*, Department of Psychology, Catholic University of Milan, Italy

**Keywords:** PTSD, re-offending risk, worry, perceived social support, childhood abuse and neglect

## Abstract

**Background:**

Studies of posttraumatic stress disorder (PTSD) are mainly focused on victims of trauma. Very few studies explored the links between PTSD symptoms and re-offending risk in perpetrators of violence.

**Objective:**

The aim of the study was to assess the effect of PTSD symptoms on re-offending risk in prisoner populations with a focus on indirect effects of worry and a negative perception of other people’s support on the relationship between PTSD and re-offending risk.

**Methods:**

75 prisoners (25 females, mean age: 44.36 years; 50 males, mean age: 34.7 years) were assessed for exposure to child abuse and neglect, PTSD symptoms, worry, a negative perception of other people’s support and re-offending risk. Mediation analyses tested the indirect effects of worry and a negative perception of other people’s support on the relationship between PTSD and re-offending risk.

**Results:**

72% participants presented PTSD symptoms and 30.7% were at risk of re-offending. Mediation analyses supported the hypothesis of a mediation pathway from PTSD to worry and a negative perception of other people’s support to an increased risk of re-offending.

**Conclusions:**

The results indicate that prisoners report high rates of PTSD symptoms; furthermore, they highlight an important relationship between PTSD and re-offending risk. Findings suggest that future research should test further the indirect effects of negative cognitive and emotional states on the relationship of PTSD and re-offending risk and explore more in depth the role of PTSD to assess and treat prisoners.

Offenders are very often exposed to complex histories of trauma (Ardino, 2011Ardino, 2012; Garbarino, 1995): child abuse and neglect, poverty, sexual molestation, witnessing violence are among most common risk factors for posttraumatic reactions concomitant with antisocial behaviour (Dziuba-Leatherman & Finkelhor, 1994). In 1989, a pioneering study by Widom demonstrated a clear link between traumatic exposure and antisocial behaviour, showing that individuals with an experience of abuse prior to the age of 11 were at a greater risk of being arrested in adolescence (Maxfield & Widom, 1996). The study has been corroborated by more recent findings (Erwin, Newman, McMackin, Morrisey, & Kaloupek, 2000; Foy, Ritchie, & Conway, 2012; McGruder-Johnson, Gleaves, Stock, & Finch, 2000) suggesting that posttraumatic stress disorder (PTSD) is significantly higher in the prison population than in community samples (Haapasalo & Pokela, 1999; Wright, Borrill, Teers, & Cassidy, 2006). A review on adolescent offenders by Vermeiren (2003) highlighted rates of PTSD ranging from 4% (Richards, 1996) to 65% lifetime PTSD (Cauffman, Feldman, Waterman, & Steiner, 1998). A more recent systematic review by Goff, Rose, Rose, and Purves (2007) found a prevalence of PTSD in adult sentenced prisoners ranging from 4% (Brink, Doherty, & Boer, 2001) to 21.4% (Butler, Levy, Dolan, & Kaldor, 2003). Maschi et al. investigated PTSD symptoms in older adults in prison revealing that younger participants reported higher levels of cumulative traumatic and stressful life events (Maschi, Morgen, Zgoba, Courtney, & Ristow, 2011).

Incarcerated women are, often, victims of a complex history of trauma; the interrelationship among trauma, their offending patterns, and related mental illness plays a major role in determining their health outcomes and risk of recidivating (Harner & Burgess, 2011). Prevalence studies of PTSD among male inmates are very rare. In their study, Akyüz, Kuğu, Sar, and Doğan (2007) examined childhood abuse, dissociation and PTSD in prisoner men highlighting a lifetime PTSD of 66.4% and more frequent dissociative symptoms than in the general population. A recent study analysing gender differences in trauma exposure and PTSD symptoms showed that prisoner males reported higher rates of witnessing harm to others in childhood and prisoner females presented higher rates of interpersonal trauma (Komarovskaya, Booker Loper, Warren, & Jackson, 2011).

The above findings outlined a high prevalence of PTSD in incarcerated populations; very rarely, however, available studies explored the effect of PTSD on re-offending risk. Furthermore, studies of the indirect effects of negative repetitive thinking on such relationship are scant. This study aims to explore the effect of PTSD symptoms on re-offending risk focusing on the mediating role of two forms of negative repetitive thinking—worry and a negative perceptions of other people's support—on the relationship PTSD-re-offending risk.

## PTSD and re-offending risk: worry and a negative perception of other people's support

Repetitive negative thinking—like worry and rumination—increases vulnerability to multiple psychiatric disorders, including PTSD (McEvoy, Watson, Watkins, & Nathan, 2013; Michael, Halligan, Clark, & Ehlers, 2007). Being conceptualised as a failure of emotion regulation (Rottenberg & Gross, 2007), rumination and worry respectively include thoughts about the past (Ehlers & Clark, 2000) and thoughts about the future (Borkovec, Ray, & Stöber, 1998; Borkovec, Robinson, Pruzinsky, & De Pree, 1983). Both forms of such negative thinking impair problem-solving and erode healthy information processing (Nolen-Hoeksema, Wisco, & Lyubomirsky, 2008).

Literature provides wide evidence that rumination predicts PTSD symptom severity (Clohessy & Ehlers, 1999; Ehlers, Mayou, & Bryant, 2003; Murray, Ehlers, & Mayou, 2002; Steil & Ehlers, 2000). In offender populations, rumination can incite an increase in negative emotions and deter prosocial responses such as empathy (Witvliet et al., 2008). Furthermore, a couple of studies investigated rumination and its role in trauma-related intrusive memories in prisoners (Evans, Ehlers, Mezey, & Clark, 2007a, 2007b), highlighting its key role in processing the offence.

Re-offending risk is always contingent to a series of factors. To reduce such a risk, a cognitive change is required by the presence of automatic thoughts and as a consequence of anger-like rumination (Simpson & Papageorgeou, 2003). In the forensic literature, anger is usually seen as a subjective emotional state that culminates from perceptual and other cognitive processes reciprocally interacting with physiological arousal in response to environmental cues (Novaco, 2003). Rumination over anger provoking situations affects the degree of control an individual has over their anger increasing the risk of re-offending, if not addressed. Research found that rumination over anger was related to sexual recidivism in groups of mixed child molesters (Hudson, Wales, Bakker, & Ward, 2002; Thornton, 2002). Whilst rumination may be well implicated in the risk of recommissioning offences and a growing body of evidence is pointing the central role of negative emotional states in the offence process (Day, 2009), less is known about the role of worry as a mediator between PTSD and risk of re-offending—conceiving worry as a strategy to control perceived threat and to lessen anxiety (Borkovec, Alcaine, & Behar, 2004; Papageorgiou & Wells, 2003; Wells & Sembi, 2004) and as a form of cognitive avoidance which inhibits emotional regulation (Borkovec et al., 2004).

Literature on perceived social support (PSS) suggests that PSS mediates the links between stressful life events and psychological consequences (Richmond, Ross, & Egeland, 2007). Furthermore, a person's perception of the availability of others as a resource, rather than actual support received, plays important roles in coping effectiveness when facing distress. Trauma literature sheds light on the role of social support as a protective factor against the development of PTSD (Brewin, Andrews, & Valentine, 2000; Ozer, Best, Lipsey, & Weiss, 2003) and demonstrates that social support mediates the association between PTSD and other factors across a variety of samples (Yap & Devilly, 2004). For example, less social support and lower availability of secure relationships mediated the relationship between PTSD and poor social functioning in a sample of Iraq and Afghanistan veterans (Tsai, Harpaz-Rotem, Pietrzak, & Southwick, 2012). In another study, the appraisal of social support was a significant moderator of types of trauma and physical injury in victims of motor vehicle accidents (Gabert-Quillen et al., 2012). Kaniasty and Norris (1992) reported that PSS buffered symptoms of depression, anxiety, fear and hostility in victims of violent crime. Another study of women who experienced partner violence revealed that social support perceived by the victims was a potent mediator of the violence–distress association (Thompson et al., 2000).

Forensic research suggests that support of family and friends and social community investment are well known protective factors to prevent antisocial behaviour (Laub & Sampson, 2003; Stouthamer-Loeber, Wei, Loeber, & Masten, 2004). There are also several studies attesting the positive effect of an available social support in prisoner populations (Harp, Oser, & Leukefeld, 2012; Jiang, Fisher-Giorlando, & Liping, 2005). The experience of incarceration also encourages a negative perception of other people's support as a result of being socially excluded and/or stigmatized for the offence. Furthermore, other studies suggest that social support lessens the level of anger providing evidence on the relationship between posttraumatic anger and social support in a group of prisoners (Schützwohl & Maercker, 2000). What remains unknown, however, is how a negative perception of other people's support mediates the relationship between PTSD and re-offending risk.

Risk assessment instruments for the prediction of offending over time rarely consider the complexity of posttraumatic aspects in the evaluation of prisoner populations. It has become increasingly clear that a more detailed investigation of the nature of predictors of risk is crucial in a more accurate management of re-offending risk (Dempster & Hart, 2002; Douglas & Kropp, 2002), including an accurate understanding of the relationship between PTSD symptoms and criminal behaviour. In a trauma-informed perspective, the precise nature of such a relationship should be explored in conjunction with risk factors currently discussed in the literature—static vs. dynamic factors, namely unchangeable determinants (e.g., static factors, such as previous violent behaviour, age at first conviction, gender) and changeable determinants (e.g., dynamic factors, such as ongoing psychological distress or psychopathology).

In this framework, a better understanding of the indirect effects of worry and a negative perception of other people's support on relations between PTSD and re-offending risk could represent an important advance in the field. Worry and a negative perception of other people's support may increase emotion dysregulation, anger, and emotional withdrawal, amplifying associations between PTSD symptoms and risk of re-offending. This paper hypothesises a mediation effect of worry and a negative perception of other people's support predisposing prisoners with PTSD to an exacerbation of their symptoms, and these symptoms then translate into poorer emotion regulation and, thus, to an increased risk of re-offending. We conceptualised worry and a negative perception of other people's support as two mediating variables on the basis of our interpretation of the extant literature on both PSS and worry in PTSD and offending behaviour. To test the hypothesis, we used the Preacher and Hayes (2008) model of simple mediation based on bootstrapping techniques for non-parametric samples.

## Methods

### Participants

Participants were recruited from three prisons in Northern Italy, hosting 350 prisoners in total. After a presentation of the study, 95 prisoners agreed to participate in the study. A group of prisoners was excluded from the study according to specific exclusion criteria (e.g., a serious ongoing mental illness and current psychiatric medication). The final sample was composed of 75 prisoners (25 females, mean age = 44.36 years, *SD*=12.27; 50 males, mean age = 33.98 years, *SD*=7.65), who committed a range of medium to serious violent offences. Medium violent offences were robbery with threat, whilst serious violent crimes included homicide or serious assault or injuries to others. Demographic characteristics of the sample are described below in Table 1.

**Table 1:** Demographic characteristics of the sample

	Males		Females		Total	
			
	*N*	%	*N*	%	*N*	%
Marital status						
Married	3	12.0	9	18.0	12	16.0
Single	11	44.0	31	62.0	42	56.0
Divorced	9	36.0	6	12.0	15	20.0
Missing	2	8.0	4	8.0	6	8.0
Education						
Elementary school	1	4.0	8	16.0	9	12.0
Secondary school	14	56.0	35	70.0	49	65.3
High school	6	24.0	6	12.0	12	16.0
Graduate degree	2	8.0	–	–	2	2.7
Missing	2	8.0	1	2.0	3	4.0

The study adhered to prison ethics guidelines according to which prisoners must be prevented from experiencing any research-related distress. Therefore, participants were given the possibility of not being involved in the study. Prisoners who chose not to participate in the study reported that they made their decision because they feared a potential delay of their release or resulting more complicated with prison officers and other prisoners.

### Measures

Measures were selected according to the research aims and hypothesis. Selected self-report questionnaires assessed childhood abuse and neglect, PTSD symptoms, worry, a negative perception of other people's support and re-offending risk.

#### Childhood abuse and neglect

The Childhood Experience of Care and Abuse Questionnaire (CECA-Q) (Bifulco, Bernazzani, Moran, & Jacobs, 2005) is a questionnaire focusing on the period prior to age 17. It records basic demographic material and childhood information on family arrangements and parental loss to identify the relevant parent figures raising the child. *Physical* and *sexual abuses* are introduced with screening questions, while *antipathy* (a pathological form of parental criticism) and *neglect* are measured by scales repeated for mother and father figures. A *care* score is derived from antipathy and neglect, indicating a lack of good parenting. Cronbach's α in this sample was .89 for maternal care and .94 for paternal care. Original Cronbach's α for CECA-Q ranged between .80 and .81

#### PTSD symptoms

The Los Angeles Symptoms Checklist (LASC) (King, King, Leskin, & Foy, 1995) is a 43-item self-report questionnaire, which measures current PTSD. Seventeen of the items correspond to the three symptom clusters B, C, D, according to DSM-IV-TR diagnostic criteria (APA, 2000). Each item is rated on a five-point scale ranging from 0 (not a problem) to 4 (extreme problem). Scores can range from 0 to 172. The LASC can be administered in 10–15 min. To be considered a positive PTSD case, a respondent must endorse an appropriate combination of symptoms with a rating of two or higher. A partial PTSD diagnosis may be considered if a respondent endorses two of the three criteria. The LASC also provides a score for PTSD severity by summing the scores of the 17 items reflecting PTSD symptoms. The sum of all 43 items provides a global assessment of distress and interference related to traumatic exposure. The cut-off score for PTSD is 56. Original Cronbach's α for LASC is .95; in this sample, Cronbach's α was .93.

#### Worry and negative perception of other people's support

The Penn State Worry Questionnaire (PSWQ) (Meyer, Miller, Metzger, & Borkovec, 1990) is a 16-item self-report questionnaire, which assesses an individual's general tendency to worry excessively without reference to specific content of the worries. Each item presents a statement and is followed by a five-point Likert-type response scale representing how typical the individual feels the statement is of him or her. In scoring the PSWQ, a value of 1, 2, 3, 4, and 5 is assigned to a response depending upon whether the item is worded positively or negatively. Items, 1, 3, 8, 10 and 11 are reverse scored. The total score of the scale ranges from 16 to 80. PSWQ has shown an average Cronbach's α of .91 and, across intervals from 2 to 10 weeks, an average test–retest correlation of .84 (Molina & Borkovec, 1994; Stöber, 1995). In this sample, Cronbach's α was .85.

The perception of others’ responses (Dunmore, Clark, & Ehlers, 1999, 2001) is a 13-item self-report questionnaire (α=.91) assessing negative perceptions of other people's people support (i.e., “People who I thought would stand by me have let me down”) and seven items (α=.89) regarding positive perceptions of others’ reactions (“Other people are genuinely concerned about me”). For this research, only the 13-item negative perception subscale was used. Scores were obtained by the sum of each item. Cronbach's α for Social Support Scale—negative perception in this sample was .85.

#### Re-offending risk

The Inventory of Offender Risk, Needs, and Strengths (IORNS) (Miller, 2006) is a 130-item true/false self-report measure for the assessment of risk, dynamic needs, and protective strengths. The Static Risk Index (SRI) consists of 12 items that assess unchangeable/historical factors related to re-offence. The Dynamic Need Index (DNI) consists of 79 items on the six-dynamic need scales: Criminal Orientation, Psychopathy, Intra/Interpersonal Problems, Alcohol/Drug Problems, Aggression, and Negative Social Influence. The Protective Strength Index (PSI) consists of 26 items and is the sum of the Personal Resources and Environmental Resources scales. Higher scores on the protective strength scales and index indicate more strength or protective influence in that area. The IORNS includes a fourth index, the Overall Risk Index (ORI), which takes into account the three focal areas assessed. Items scores—ranging from 0 to 1—must be converted to *T* scores and percentiles by age and gender criteria. Cronbach's α for IORNS main scales in this sample was .85 and for IORNS subscales was .85. Original Cronbach's α for IORNS ranged between .91 and .73.

### Procedure

In all three institutions, researchers invited prisoners to an introductory meeting to learn about the research. Prisoners had the opportunity to ask questions about the study and to have adequate time to decide whether they wanted to be involved in the research. At assessment time, prison officers accompanied each participant to a quiet room in the prison ward where participants completed the self-report questionnaires. Overall, each participant needed 40 min to complete the questionnaires. Questionnaires were collected by the psychology unit staff and then locked in a safe room in their offices to maintain confidentiality. Each participant was assigned a code; furthermore, researchers remained blind about the identity and type of offence the participants had committed. Upon termination of the study, researchers had a meeting with all participants to present study findings. A written report was also provided to the Italian Ministry of Justice.

### Ethical considerations

The Italian Ministry of Justice and the Research Committee of the three institutions participating in the study cleared the research. Participants were informed about their rights on at least three occasions: (1) first contact, (2) oral information prior to the administration, and (3) a written information sheet. Anonymity and confidentiality were ensured through a code attribution to each participant so that no names were used. Furthermore, the participants were informed about their right to withdraw from the study at any time.

### Data analysis strategy

Data were analysed with a three-step strategy. Firstly, descriptive statistics were performed; secondly, correlational analyses were conducted to explore existing relationships among study variables and to gain information to develop the mediation model. Finally, mediation analyses used bootstrap procedures, more appropriate for non-parametric samples using an application for SPSS (Preacher & Hayes, 2008), which includes a formal test of the magnitude of any indirect effects of worry and a negative perception of other people’ support on the relationship PTSD-re-offending risk. The model shows how the causal effect of PTSD (independent variable) can be apportioned into its indirect effect on re-offending risk (dependent variable) through worry and a negative perception of other people's support (mediators) and its direct effect on the named dependent variable. Usually, calculations are based on non-standardised regression coefficients. This approach was chosen over Baron and Kenny's (1986) causal steps approach because it (1) provides a direct test of indirect (or mediating) effects, (2) is a more sensitive test of mediation, and (3) reduces the opportunity for incorrect conclusions amplified by the multiple significance tests required to perform a series of regression.

## Results

### Descriptive statistics

#### Incidence of childhood abuse and neglect

The majority of participants experienced childhood abuse and neglect (neglect, emotional, physical and sexual abuse), determined against cut-off scores previously identified for the CECA-Q questionnaire (Bifulco et al., 2005). Participants experienced paternal neglect (40%) and lack of paternal care (36%), which results from a combined score of antipathy and neglect. Twenty-eight percent of participants experienced maternal physical abuse whereas 26.7% experienced paternal physical abuse. Finally, 14.7% of the sample had a history of sexual abuse. When the variable of gender was controlled, paternal antipathy was the most disruptive experience for female prisoners (28%; χ^2^=2.97; *p <* .08); conversely, most male prisoners experienced paternal neglect (50%; χ ^2^=6.25; *p <* .05). Consistent with the literature, a greater number of women (36%) suffered from sexual abuse; only 4% of prisoner men were sexually abused (χ ^2^=13.63; *p <* .001). By contrast, male prisoners experienced more paternal physical abuse than female prisoners (30% vs. 20%).

#### Incidence of re-offending risk

Cut-off scores of IORNS were used to assess re-offending risk. A total of 30.7% of the sample scored significantly higher than the cut-off score (see Table 2 below). Findings have also shown that women were at a lower risk of re-committing a crime (24%; *n*=6) than men (34%; *n*=17). The χ ^2^ was not significant in this case.

**Table 2:** Frequency of prisoners with high re-offending risk as measured by IORNS

Re-offending risk	Total *N* (%)	Females *N* (%)	Males *N* (%)
Above cut-off	23 (30.7)	6 (24.0)	17 (34.0)
Under cut-off	52 (69.3)	19 (76.0)	33 (66.0)
Total	75	25	33

*N*=75; IORNS: Inventory of Offender Risks, Needs and Strengths.

#### Incidence of PTSD

A global score and a partial score of all clusters of PTSD symptoms were calculated. Of the total sample, 72% (*n*=54) met diagnostic criteria for PTSD. In terms of gender differences, 60% (*n*=15) of females met PTSD criteria, compared to 78% (*n*=39) of males; this difference was not significant (χ ^2^=2.67; n.s.). Since the diagnosis of PTSD was obtained via a self-report questionnaire, the scores could not be verified with a clinical interview. Therefore, data should be interpreted cautiously and participants should be considered as having a “probable PTSD.” Furthermore, the onset of PTSD may have been determined by other traumatic events that occurred over the life course, a potential confounding factor that could not be controlled in this study.

#### Incidence of worry and negative perception of other people's support

A cut-off score of 45 was used to discriminate the sample according to the level of pathological worry (Meyer et al., 1990). Forty-eight percent (*n*=36) of participants presented a pathological tendency to worry. Men (52%; *n*=26) presented higher levels of worry than women (40%; *n*=10); this difference was not significant (χ^2^=.96; n.s.). Fifty-two participants completed the perception of others’ responses scale assessing the negative perception of other people's support (*M*=22; *SD*=11) demonstrating a tendency to have a negative perception of other people's support.

### Correlation analyses

#### Re-offending risk and PTSD

A series of two-tailed Pearson linear correlations was conducted to test relationships among target variables. The first analysis tested the correlation between re-offending risk and PTSD symptoms; results showed a strong correlation between PTSD symptoms and re-offending risk (see Table 3).

**Table 3:** Pearson correlations between PTSD symptoms, and re-offending risk

	IORNS SRI r	IORNS DRI r	IORNS PSI r	IORNS TOT r
LASC severity	.22	.40**	−.16	.33
LASC global	.25	.56**	−.34*	.52**
LASC B “re-experiencing”	.09	.29	−.18	.21
LASC C “avoidance”	.25	.46**	−.11	.43**
LASC D “hyperarousal”	.15	.28	−.11	.23

*N*=75; LASC: Los Angeles Symptoms Checklist; IORNS: Inventory of Offender Risks, Needs and Strengths; PTSD: posttraumatic stress disorder; SRI: Static Risk Index;

* *p*<.05

** *p*<.01; α corrected with a Bonferroni test=.003.

The dynamic risk index was correlated with PTSD severity and PTSD symptoms of avoidance. The PSI was inversely correlated with PTSD global score, corroborating further the role of PTSD in the maintenance of re-offending risk. Overall, correlations supported the study hypothesis of a relationship between PTSD and re-offending.

#### Childhood abuse and neglect and PTSD

Findings showed no relationship between childhood abuse and neglect perpetrated by the mother and PTSD. Conversely, data showed a strong association between symptoms of re-experiencing and paternal physical abuse. Both paternal antipathy (*r*=.394; *p <* .01) and paternal lack of care (*r*=.423; *p <* .01) correlated with re-experiencing symptoms. These results may indicate that paternal poor parenting could be a better predictor of PTSD development than poor maternal care in this population.

#### Worry, negative perception of other people's support and PTSD

Worry and the negative perception of other people's support were correlated with PTSD symptoms (see Table 4).

**Table 4:** Pearson correlations between PTSD symptoms and worry and negative social support

	PSWQ	Negative social support
LASC severity	.57**	.42**
LASC global	.58**	.48**
LASC B “re-experiencing”	.47**	.47**
LASC C “avoidance”	.34	.34
LASC D “hyperarousal”	.55**	.41**
Perceived negative social support	.07	−

*N*=75; LASC: Los Angeles Symptoms Checklist; PSWQ: Penn State Worry Questionnaire; PTSD: posttraumatic stress disorder;

** *p*<.01; α corrected with a Bonferroni test=.003.

Table 4 demonstrated a strong relationship between worry and re-experiencing and hyperarousal symptoms of PTSD; additionally, worry was correlated with the global level of distress measured by the LASC. Negative perception of other people's support correlated with re-experiencing and hyperarousal symptoms, and with the level of global distress. All correlations were also corrected using a Bonferroni test (corrected α=.003) due to the great number of simultaneous correlations.

### Mediation analyses

The total effect of PTSD (independent variable) over dynamic re-offending risk was significant (β=.36; s.e.=.78; *p <* .001). The effect of the independent variable through the mediators (Worry, Perceived Social Support), or the “c prime path,” is lower (β=.23; s.e.=.97; *p <* .05). The mediation model is significant and explains a good amount of variance (R^2^=.41; *p <* .001). Using bootstrapping, the simple indirect effect was significant, as indicated by 95% confidence intervals around the indirect effect that did not contain values of zero (see Table 5).

**Table 5:** Indirect effects of worry and negative social support on dynamic re-offending risk

Mediator	Estimate (SE)	*Z*	95% confidence lower interval	95% confidence upper interval
Worry	.06 (.04)	1.26	− .02	.14
Negative social support	.11 (.04)	2.33**	.02	.21

Bootstrapping (10,000 samples) mediation analysis.

** *p*<.01.

Findings are consistent with the presence of an indirect effect from PTSD to re-offending risk through worry and a negative perception of other people's support confirming, therefore, the hypothesis of this study.

## Discussion

The study investigated the relationship between PTSD and re-offending risk with a focus upon the indirect effects of worry and a negative perception of other people's support on such relationships. Descriptive statistics showed that childhood abuse and neglect rates and PTSD symptoms were higher than in community samples. Results confirmed outcomes of previous studies (Abram et al., 2004; Brinded, Simpson, Laidlaw, Fairley, & Malcolm, 2001; Vermeiren, 2003). A study, for example, documented a lifetime prevalence rate for PTSD among incarcerated men at 33% (Ehlers, Maerker, & Boos, 2000), more than four times higher than the rate for men in the general population. Existing literature has widely demonstrated that prisoner women have extensive histories of trauma; the study also showed that males experienced complex trauma in their childhood. Men reported more extensive experiences of paternal physical abuse and higher rates of PTSD compared with previous studies on prisoner men (Breslau, Peterson, Poisson, Schultz, & Lucia, 2004; Kessler, Sonnega, Bromet, Hughes, & Nelson, 1995; Perkonigg, Kessler, Storz, & Wittchen, 2000). Psychological abuse was the most frequent adverse experience. More specifically, paternal antipathy was the most common distressing experience reported by female prisoners whereas male prisoners reported most frequently a lack of paternal care and paternal neglect. The study highlighted the effect of emotional abuse and neglect in the history of prisoners indicating the need for further research to explore different trajectories and outcomes of abusive parenting linked to criminal behaviour. Findings have also highlighted significant levels of chronic worry and of a tendency to have a negative perception of other people's support. Results corroborated previous studies conducted by Evans and colleagues in which 36% of the participants described rumination about their violent offence and reported ruminating for several hours per week (Evans et al., 2007a, b).

Correlational analyses presented interesting results. They showed a strong relationship between paternal psychological abuse and PTSD, indicating the need to investigate the role of psychological maltreatment more closely. Data showed a strong association between symptoms of re-experiencing and paternal physical abuse along with significant correlations between paternal antipathy and lack of care with re-experiencing symptoms highlighting an important contribution of paternal neglect in the histories of prisoners. Significant correlations were also found between worry, a negative perception of other people's support and PTSD symptoms.

Data highlighted a strong association between PTSD symptoms and the risk of re-offending. IORNS overall index of risk correlated with PTSD global scores. Furthermore, IORNS Protective Strength Index was inversely correlated with PTSD, further supporting the hypothesis of the study that predicted a relationship between PTSD and risk factors of re-offending.

### Mediation analyses

Examination of specific paths within the simple indirect effects indicated that higher levels of pre-existing negative emotionality (worry and a negative perception of other people's support) predicted higher levels of re-offending risk in prisoners with PTSD symptoms. This is consistent with existing literature suggesting that negative emotionality creates vulnerability for more severe symptoms of PTSD (Rubin, Boals, & Bernsten, 2008).

PTSD symptoms were, then, significantly associated with both higher worry and a negative perception of other people's support. The novelty of this study may be particularly important to reflect on the nature of distressing emotional states and may partially explain why PTSD symptoms are interlinked to re-offending risk through a complex cascade of effects.

Furthermore, findings partially corroborate existing studies. In a study on Vietnam veterans, Boscarino (1995) investigated the link between PTSD, social support and psychiatric problems including generalized anxiety, depression, drug abuse and alcohol abuse. In such a study, the level of social support was linked with current PTSD, generalized anxiety, current depression, and current alcohol abuse. According to Dunmore et al. (1999), when individuals perceive to be treated negatively by others they also perceive an ongoing harm from their everyday social world. In line with the results, it could be speculated that the unprocessed trauma may lead to a higher re-offending risk rate via an interpretation of the world as a stance against the offender. Moreover, the interplay between PTSD and a negative perception of other people's support may impede the acceptance of the trauma and a healthy emotional processing.

Furthermore, the negative perception of other people's support and worry may be engendered by the impact of imprisonment on offenders. Imprisonment is known to be a stressful life event (Kupers, 1999) and the separation from the social support network may contribute to a high state of anxiety (Paulus & Dzindolet, 1993). The effects of incarceration could be more distressing for prisoners with posttraumatic symptoms who may have more difficulties to adjust as a result of their disorder (Islam-Zwart, 2004) and who may be more likely to perceive imprisonment as threatening and as a trigger of their posttraumatic symptomatology. The mediation analysis encourages a deeper understanding of the complex relationship between a history of trauma and its consequences and re-offending risk.

### Limitations of the study

Results should be interpreted with several limitations in mind. The retrospective nature of this study renders more problematic to determine the accuracy of childhood abuse and neglect experienced by participants, including the types of events and ages of occurrence, and posttraumatic symptomatology. Furthermore, the Italian regulations about confidentiality prevented us gathering information about specific offences committed by participants.

A second limitation regards the sample size; there was a great disparity between males and females that may have confounded the final results. Moreover, participants were recruited on a voluntary basis and in specific prisons in Northern Italy; therefore, the generalizability of the results may be questionable. The distribution of the sample is *not* normative in terms of severity of previous traumatic experiences, which led to a very high prevalence rate of PTSD. Such finding should be cautiously interpreted and other variables should be considered, such as the lack of trauma-focused treatments in the prison settings or—in some instances—by the difficult living conditions in prison settings. Prison living conditions may have worsened the symptoms of PTSD and may have functioned as an ongoing trigger preventing the usual adjustment to imprisonment. Furthermore, the self-selection bias, which could not be controlled, may have further jeopardized some of the findings.

A third limitation concerns the nature and scope of traumatic experiences under investigation. The study analysed only the prevalence of childhood abuse and neglect perpetrated by caregivers; for this reason, it was not possible to control for other potential traumatic events that may happened later in life to the same individual, their combined effects with childhood abuse and neglect and their link to PTSD or criminal behaviour. For example, literature showed a strong association between witnessing violence, trauma and later offending. In their study, Eitle and Turner (2002) indicated that recent exposure to violence in the community along with a history of receiving traumatic news, recent stressful life events, and associations with criminal peers increase the risk of offending. Future studies should explore the effects of extrafamilial traumatic events on offending behaviour and re-offending risk. Finally, given their prolonged exposure to complex trauma, the study should have also considered symptoms of complex PTSD and comorbid disorders. Several studies, for example, have shown that perpetrators of crime are at higher risk of substance abuse than community samples (Chiles, Von Cleve, Jemelka, & Trupin, 1991). Another study has also shown a high frequency of personality disorders (Davison, Leese, & Taylor, 2001). Future studies should better explore the dual role of PTSD and other disorders in favouring and maintaining criminal behaviour and a high risk of re-offending.

The aforementioned limitations suggest that future studies should expand the sample, control for the type of criminal offence committed and assess the effect of imprisonment and the offence as PTSD-inducing life events.

As for worry and a negative perception of other people's support, there were two limitations. The first limitation concerns the analysis of worry: depressive and angry rumination were not investigated. Probably, ruminative-like thoughts about the past are also occupying offenders’ minds as other studies have demonstrated (Evans et al., 2007a, b). Investigating this aspect could have better highlighted offenders’ views of their past and possibly the link between trauma and re-offending. The second limitation concerns the perception of social support: the study only investigated the negative perception of others. This may have overshadowed the possible positive effects of a positive perception of others. In further studies it could be important to counterbalance the negative perception of others with a measurement of the positive aspects of PSS.

Despite several limitations, the study made an important contribution to the understanding of the role of PTSD in maintaining re-offending risk in prison populations. It also shed light on the role of negative emotional states as mediators of the PTSD and re-offending relationship. Future studies should concentrate on the absence of follow-up studies, at extended intervals investigating further the relationship between PTSD and re-offending risk. In fact, on one hand, PTSD is generally a disorder of long duration and, on the other hand, re-offending risk is affected by a patterned set of events, with the timing, nature, and characteristics of initial events and experiences influencing subsequent events. Identifying patterns in the timing of offending and PTSD course and investigating whether there is overlap among these patterns is an important theoretical and practical endeavour as the empirical knowledge based on this question is virtually non-existent.

Another future area of research could be the investigation of indirect effects over offence-related PTSD symptoms. According to Harry and Resnick (1986) and Pollock (1999), committing a violent offence may lead to the development of secondary posttraumatic reactions. Some recent studies on selected populations have suggested that the commission of a violent crime may lead to PTSD (Kruppa, Hickey, & Hubbard, 1995; Spitzer et al., 2001). In a recent study, Crisford, Dare, and Evangeli (2008) found that higher levels of trauma-related symptoms were associated with higher levels of guilt cognitions related to the offence. Furthermore, Gray et al. (2003) have partialled out types of violent offences to measure PTSD rates in inmate populations and found that the more life threatening the offence was, the more likely offenders suffered PTSD symptoms.

Offender rehabilitation programmes seek to reduce re-offending risk. They target dynamic risk factors (Andrews & Bonta, 1998; Hanson & Harris, 2000), namely changeable risk factors that are amenable to change through intervention. Despite the high prevalence of PTSD and, therefore, the obvious need for effective trauma treatment programmes in prison settings, evidence supporting what would work best for traumatized prisoners is still at its infancy. The study highlighted the importance of evaluating the eventual feasibility, acceptability and efficacy of trauma therapy as an adjunct to treatment-as-usual in prison. Therefore, clinical trials to test the impact of trauma-focused interventions on re-offending risk rates and to implement trauma-informed services in the prison system are warranted. The use of a trauma-based framework may offer an effective support to identify risk factors for future violent offending, specifically aiming to reduce the risk of violent re-offending. Further, the inclusion of a more sensitive PTSD evaluation in re-offending risk assessment may help to understand the behaviours that prison staff must manage and see their role in future events.

## Conclusions

In conclusion, the study contributed to the field by better highlighting the mechanisms that link PTSD to re-offending risk. Furthermore, the study pointed out the importance of considering the indirect effects of negative emotional states to explore further the complexity of re-offending risk.
